# New Dihydro-β-agarofuran Sesquiterpenes from *Parnassia wightiana* Wall: Isolation, Identification and Cytotoxicity against Cancer Cells

**DOI:** 10.3390/ijms150611111

**Published:** 2014-06-20

**Authors:** Chao Lv, Zuo-Lue Zheng, Fang Miao, Hui-Ling Geng, Le Zhou, La-Ping Liu

**Affiliations:** 1 College of Science, Northwest A&F University, Yangling 712100, China; E-Mails: dear.chao.lu@gmail.com (C.L.); zhengzuolue@gmail.com (Z.-L.Z.); genghuiling5@163.com (H.-L.G.); 2 College of Life Science, Northwest A&F University, Yangling 712100, China; E-Mail: miaofangmf@163.com; 3 College of Food Science and Engineering, Northwest A&F University, Yangling 712100, China

**Keywords:** dihydro-β-agarofuran, sesquiterpene, *Parnassia wightiana*, cytotoxicity

## Abstract

Five new (**4**–**8**) and three known (**1**–**3**) dihydro-β-agarofuran sesquiterpene polyesters were isolated from the whole plants of *Parnassia wightiana*. The structures of all compounds were elucidated through spectroscopic analysis including 2D-NMR and HR-MS. The absolute configuration of these compounds was established by X-ray diffraction analysis, comparison of NOESY spectra and biogenetic means. The cytotoxities of compounds **2**–**8** were evaluated *in vitro* against HL-60, SMMC-7721, A549, MCF-7 and SW480 cell lines. Compounds **5**–**7** exhibited the highest activities with IC_50_ values of 11.8–30.1 μM in most cases. The SAR revealed that the introduction of hydroxyl group was able to significantly improve the activities of the compounds for most of the cell lines.

## 1. Introduction

The sesquiterpene polyesters with a dihydroagarofuran skeleton have attracted considerable attention from synthetic organic chemists and pharmacologists due to their complex and diverse chemical structures and wide range of biological activities [1], including insect antifeedant and/or insecticidal activity [2,3], cytotoxic activity [4], antitumor [5], antitumor promoting activity [6], antitubercular [7], immunosuppressive [8], anti-HIV [9], anti-inflammatory activity [10] and reversal of the multidrug resistance (MDR) phenotype [11]. Until now, hundreds of dihydro-β*-*agarofuran compounds have been isolated from dozens of species of plants. It is noteworthy that most of the natural dihydro-β*-*agarofurans originated mainly from plants of the Celastraceae family. Only a minority were found in other family plants such as Hippocrateaceae and Lamiaceae [1]. Therefore, dihydro-β*-*agarofurans have been considered as chemotaxonomic indicators of the Celastraceae family.

*Parnassia wightiana* Wall (Saxifragaceae), commonly known as Ji-mei-hua-cao, Cang-er-qi, Qiao-mai-ye or Ding-chuang-cao, is a perennial herb and is distributed in the Qinling Mountains in China. Dried whole plants have been used as Chinese folk medicine for the treatment of leukorrhea, cough, haematemesis, carbuncle, irregular menstruation, hypertension, malaria, kidney stones, gall stones, and so on. Recently, Wang and coworkers isolated one dihydro-β-agarofuran compound from this plant, which exhibited cytotoxicity against HepG2 and MDA-10 cells and antifeedant activity against *Mythimna* *separata* larvae [12,13]. Besides that, up to now, no systematic phytochemical investigation of this plant has been reported.

**Figure 1:**
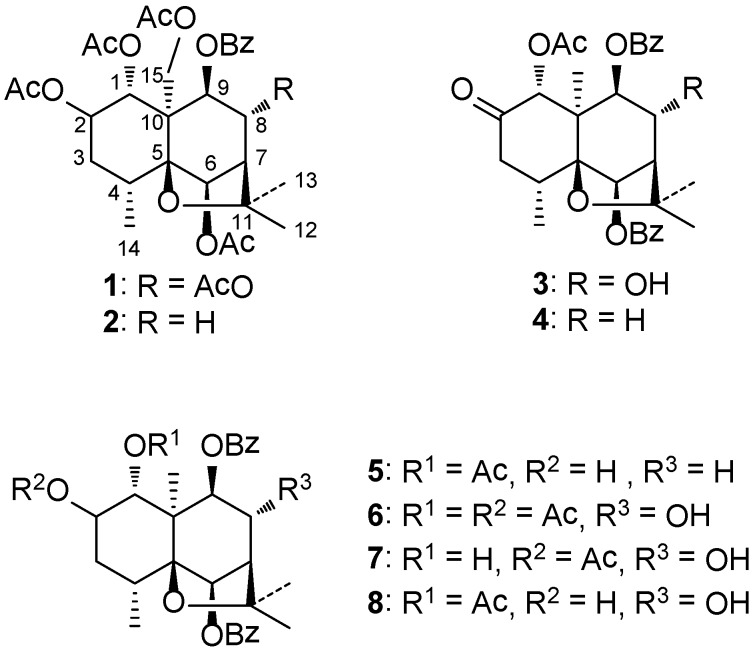
Structures of isolated compounds from *P.* *wightiana.*

In this context, this study aimed at investigating in more detail the composition, structural characteristics and bioactivity of dihydro-β-agarofuran compounds in *P. wightiana*. Herein, we described the isolation and structure elucidation of eight dihydro-β-agarofuran sesquiterpenes (Figure 1) including five new compounds (**4**–**8**) as well as their cytotoxic activity against five cancer cell lines. The structures of all compounds were elucidated by means of HR-MS, 1D- and 2D-NMR spectroscopic analysis including DEPT, ^1^H-^1^H COSY, NOESY, HSQC and HMBC experiments. Their absolute configurations were established by X-ray crystallographic analysis, biogenetic means and comparison of NOESY spectra. The isolated compounds were evaluated for cytotoxic properties against five cancer cell lines.

## 2. Results and Discussion

**Figure 2:**
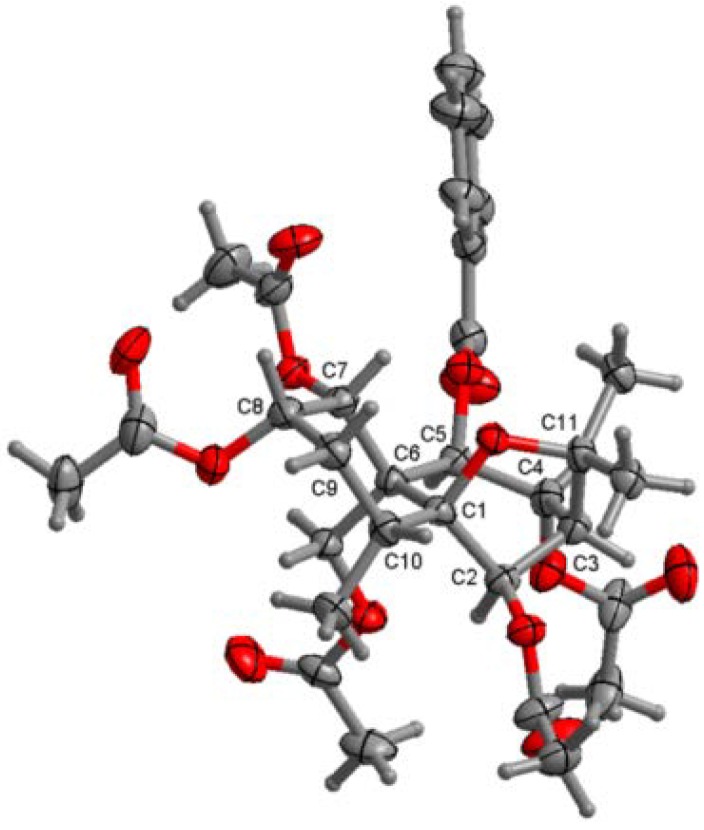
X-ray ORTEP drawing of compound **1**.

Three known compounds were identified as ejap-4 (**1**) [14], celahin B (**2**) [15,16] and 1α-acetoxy-8α-hydroxy-6β,9β-dibenzyloxy-2-oxodihydro-β-agarofuran (**3**) [12,13] by spectroscopic methods, but the previous reports did not confirm their absolute configurations. For this reason, a single-crystal X-ray diffraction experiment of **1** was performed in the present research. Its crystallographic data revealed that the torsion angle obtained corresponded to a decalin system with a *trans* union between rings A and B, of which A adopted a chair conformation and B, a half-chair conformation, whereas the furan ring presented an envelope conformation (Figure 2). Thus, **1** was elucidated as (1*R*,2*S*,4*R*,5*S*,6*R*,7*R*,8*R*,9*R*,10*S*)-1,2,6,8,15-pentaacetoxy-9-benzoyloxydihydro-β-agarofuran. Taking into account biosynthetic consideration and the same NOE effects of **2** as that of **1**, the absolute configuration of **2** can be proposed as 1*R*,2*S*,4*R*,5*S*,6*R*,7*R*,9*S*,10*R* because the only difference of **2** from **1** is the absence of acetoxyl group at C-8.

Compound **3** as a new compound from the same plant was reported by Wang *et al.* [12,13], but the authors proposed an inconsistent stereochemistry for **3**, due to the absence of the corresponding 2D-NMR analysis. In addition, they did not also provide the complete 1D-NMR data and other spectrometric data as well as physical properties. Therefore, the more detailed structure characterization of **3** is herein necessary.

Compound **3** was obtained as a white solid with [*α*]_D_^21^ +10.45° (*c* 0.331; CHCl_3 _). The ions at *m*/*z*: 551 [M + H]^+^ in ESI-MS and 573.2107 [M + Na]^+^ in HR-ESI-MS of **3** afforded a molecular formula of C_31_H_34_O_9_. The IR showed the presence of hydroxyl (3493 cm^−1^), carbonyl (1752, 1724 cm^−1^) and aromatic groups (3063, 1601 cm^−1^).

The ^13^C NMR and DEPT spectra of **3** indicated 27 carbon signals comprising three ester carbonyl carbons, one ketone carbonyl carbon, one quaternary sp^3^ carbon, three tertiary carbons including two ones linked to an oxygen atom, five secondary carbons including four ones linked to an oxygen atom, eight sp^2^ carbons including six ones linked to a hydrogen atom, one methylene carbons, and five methyl carbons (Table 1). Its NMR and mass spectra showed the presence of one acetate ester [δ_H_ 1.71 (3H, s), δ_C_ 20.0 (CH_3_CO_2_–), δ_C_ 169.5 (CH_3_CO_2_–); EI-MS *m*/*z*: 43 [Ac]^+^] and two benzoate esters [δ_H_ 7.46 (2H, t, *J* = 8.00 Hz), 7.49 (2H, t, *J* = 8.00 Hz), 7.59 (1H, t, *J* = 8.00 Hz), 7.62 (1H, t, *J* = 8.00 Hz), 8.03 (2H, d, *J* = 8.00 Hz), 8.05 (2H, d, *J* = 8.00 Hz); δ_C_ 128.6 (CH), 128.7 (C), 128.8 (CH), 129.6 (CH), 129.7 (C), 129.8 (CH), 133.6 (CH), 133.8(CH)].

Except for the signals of the ester groups, ^1^H and ^13^C NMR, DEPT and HSQC spectra showed the structural characteristics of a dihydro-β-agarofuran ketone skeleton consisting of four methyl carbons [δ_H_ 1.06 (3H, d, *J* = 7.5 Hz)/δ_C_ 18.1, 1.46 (3H, s)/δ_C_ 20.7, 1.50 (3H, s)/δ_C_ 25.6, 1.59 (3H, s)/δ_C_ 31.2 and 1.71 (3H, s)/δ_C_20.0], one methylene carbon [δ_H_ 2.29 (1H, d, *J* = 12.8 Hz) and 3.39 (1H, dd, *J* = 7.4, 12.8 Hz)/δ_C_ 44.0], six methine carbons with four linked to an oxygen atom [δ_H_ 2.72 (1H, s)/δ_C_ 55.7, 2.99–3.05 (m, quintet-like)/δ_C_ 38.8, δ_H_ 4.48 (1H, s)/δ_C_ 74.5, δ_H_ 4.97 (1H, s)/δ_C_ 80.5, δ_H_ 5.98 (1H, s)/δ_C_ 76.8, δ_H_ 6.27 (1H, s)/δ_C_ 76.1], three quaternary carbons with two linked to an oxygen atom [δ_C_ 54.8, 82.6, 89.8] and one ketone carbonyl carbon (δ_C_ 204.6).

**Figure 3:**
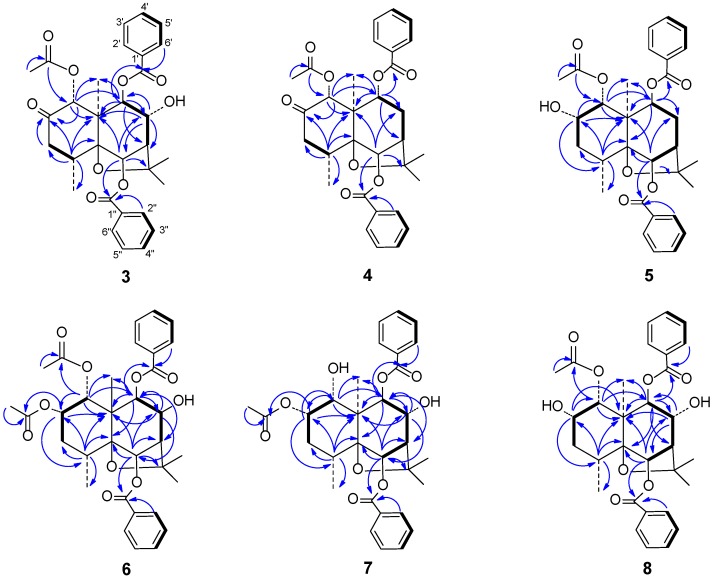
Main ^1^H–^13^C long-range correlation (→) and ^1^H-^1^H correlation (**–**) signals in the HMBC and COSY spectra of **3**–**8**.

In the HMBC spectrum (Figure 3), the correlations between δ_H_ 5.98 (s, H-1)/δ_C_ 169.5 (CH_3_CO_2_–), between δ_H_ 6.27 (s, H-6)/δ_C_ 165.5 (PhCO_2_–), and between δ_H_ 4.97 (1H, s, H-9)/δ_C_ 165.5 (PhCO_2_–) showed that the acetoxy group was at C-1 and two benzoyloxy groups were at C-6 and C-9, respectively. The correlation between δ_H_ 5.98 (s, H-1) and δ_C_ 204.6 (C=O) as well as the lowfield chemical shift and singlet of H-1 showed that the 2 position was a ketone carbonyl group. The correlations between δ_H_ 4.97 (s, H-9), 2.72 (s, H-7)/δ_C_ 74.5 (C-8) showed that the 8 site was a hydroxymethine group. The signal at δ_H_ 4.48 (s) was assigned to H-8 by the HSQC spectrum.

**Figure 4:**
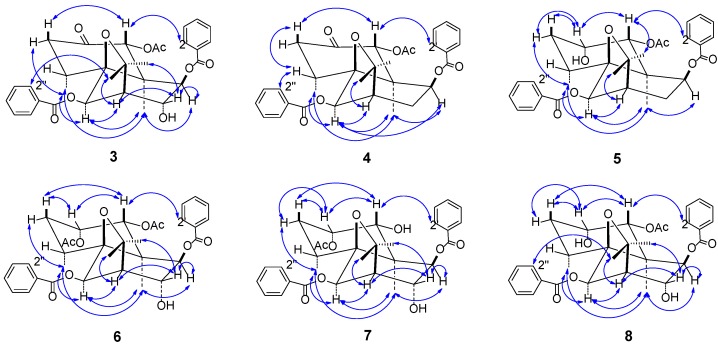
Main NOE correlation signals ( ←→ ) in the NOESY spectra of **3**–**8**.

In the NOESY spectrum (Figure 4), the correlations between δ_H_ 1.46 (s, H-15) and/1.06 (d, *J* = 7.5 Hz, H-14), 6.27 (s, H-6), 4.97 (s, H-9) showed that 6-benzoyloxy group and 9-benzoyloxy group were equatorial and axial, respectively, and the correlations between δ_H_ 5.98 (s, H-1)/3.39 (dd, *J* = 7.4, 12.8 Hz, H-3_ax_), /8.03 (d, *J* = 8.0 Hz, H-2') showed that 1-acetoxy group was equatorial. The axial assignment of 8-OH was supported by the NOE effect between δ_H_ 4.48 (H, s, H-8)/ 1.50 (3H, s, H-12). The above NOE cases were the same as that of **1**, indicating that the space orientations of the substituents in **3** were identical to that of the substituents at the same site in **1**. Thus, **3** was identified as (1*R*,4*R*,5*S*,6*R*,7*R*,8*R*,9*R*,10*R*)-1-acetoxy-6,9-dibenzoyloxy-8-hydroxydihydro-β-agarofuran-2-one.

Compound **4** was obtained as a white powder. Its molecular formula was established as C_31_H_34_O_8_ by HR-MS (*m*/*z*: 557.2144 [M + Na]^+^) and ^13^C NMR data, which had one less oxygen atom compared with that of **3**. The IR spectrum showed the presence of carbonyl (1753, 1720 cm^−1^) and aromatic (3062, 1601 cm^−1^) functionalities. The UV spectrum exhibited an absorption maximum at 244, 274, 283 nm, suggesting the existence of aromatic moieties.

The ^13^C NMR and DEPT spectra (Table 1 and Table 2) indicated that **4** had one additional methylene group and one less oxygenated methine group than **3**. The ^1^H and ^13^C NMR, EI-MS, IR and UV spectra displayed the presence of one acetate ester, two benzoate esters and one ketone carbonyl group. The ^1^H and ^13^C NMR spectra of **4** were similar to those of **3** except that the methylene group at C-8 of **4** replaced the oxygenated methine at C-8 of **3**. Thus, **4** was identified as a 1,6,9-trisubstituted dihydroagarofuran-2-one, which was further supported by the ^1^H-^1^H COSY and HMBC spectra. The complete assignments of the protonated carbons were made from the HSQC spectrum.

The carbonyl signals and regiosubstitution of three ester groups were assigned by HMBC (Figure 2). The correlations between δ_C_ 165.6 (PhCO_2_–)/δ_H_ 5.63 (s, H-6), between δ_C_ 165.2 (PhCO_2_–)/δ_H_ 5.08 (1H, d, *J* = 7.2 Hz, H-9),/δ_H_ 8.05 (d, *J* = 7.4 Hz, benzoyl-H-2′), and between δ_C_ 169.5 (CH_3_CO_2_–)/δ_H_ 6.01 (s, H-1), /δ_H_ 1.73 (s, CH_3_CO_2_–) suggested that the acetyl ester and the two benzoyl esters were positioned at C-1, C-6 and C-9, respectively.

**Table 1:** ^13^C NMR and DEPT data of compounds **3**–**8** (100 MHz).

Number	3, δ_C_^a^	4, δ_C_^a^	5, δ_C_	6, δ_C_	7, δ_C_	8, δ_C_
1	76.8, CH	77.5, CH	75.7, CH	73.0, CH	69.2, CH	75.7, CH
2	204.6, C	204.9, C	69.1, CH	70.9, CH	75.1, CH	69.0, CH
3	44.0, CH_2_	43.9, CH_2_	34.1, CH_2_	32.1, CH_2_	32.2, CH_2_	34.2, CH_2_
4	38.8, CH	38.9, CH	35.9, CH	35.8, CH	35.5, CH	35.8, CH
5	89.8, C	89.0, C	91.9, C	92.2, C	92.5, C	92.8, C
6	76.1, CH	80.0, CH	81.2, CH	77.3, CH	77.7, CH	77.5, CH
7	55.7, CH	49.0, CH	50.4, CH	57.6, CH	57.7, CH	57.7, CH
8	74.5, CH	32.5, CH_2_	32.5, CH_2_	75.2, C	75.3, C	75.3, C
9	80.5, CH	73.3, CH	75.1, CH	80.6, CH	80.1, CH	81.1, CH
10	54.8, C	55.9, C	51.3, C	50.5, C	51.8, C	50.6, C
11	82.6, C	83.7, C	84.1, C	83.4, C	83.1, C	83.1, C
12	25.6, CH_3_	26.0, CH_3_	26.5, CH_3_	26.1, CH_3_	26.1, CH_3_	26.1, CH_3_
13	31.2, CH_3_	30.7, CH_3_	31.3, CH_3_	31.8, CH_3_	32.0, CH_3_	31.9, CH_3_
14	18.1, CH_3_	18.2, CH_3_	19.6, CH_3_	19.0, CH_3_	19.1, CH_3_	19.4, CH_3_
15	20.7, CH_3_	20.0, CH_3_	21.4, CH_3_	20.9, CH_3_	19.9, CH_3_	21.6, CH_3_
AcO-1	20.0, CH_3_ 169.5, C	20.0, CH_3_ 169.5, C	20.9, CH_3_ 172.2, C	20.6, CH_3_ 171.7, C	-	20.9, CH_3_ 172.2, C
AcO-2	-	-	-	21.2, CH_3_ 172.0, C	21.5, CH_3_ 172.6, C	-
1'	128.7, C	129.2, C	130.6, CH	131.4, C	131.8, C	131.5, C
2'/6'	129.8, CH	129.8, CH	131.1, CH	131.2, CH	130.8, CH	131.1, CH
3'/5'	128.6, CH	128.5, CH	129.5, CH	129.5, CH	129.9, CH	129.5, CH
4'	133.8, CH	133.6, CH	134.4, CH	134.6, CH	134.8, CH	134.4, CH
1''	129.7, C	129.6, C	131.3, C	131.4, C	131.5, C	130.7, C
2''/6''	129.6, CH	129.5, CH	131.0, C	130.5, CH	130.5, CH	130.5, CH
3''/5''	128.8, CH	128.8, CH	129.9, CH	129.9, CH	129.6, CH	129.9, CH
4''	133.6, CH	133.5, CH	134.7, CH	134.7, CH	134.6, CH	134.6, CH
CO_2_-6	165.5, C	165.6, C	167.0, C	166.8, C	166.8, C	166.8, C
CO_2_-9	165.5, C	165.2, C	166.8, C	166.4, C	166.7, C	166.5, C

^a^ The data were acquired at 125 MHz.

In the NOESY spectrum (Figure 3), the correlations between H-15 (δ_H_ 1.34, s)/H-9 (δ_H_ 5.08, d, *J* = 7.2 Hz), H-6 (δ_H_ 5.63, s)/H-14 (δ_H_ 1.02, d, *J* = 7.5 Hz), between H_ax_-3 (δ_H_ 3.40, dd, *J* = 7.5, 12.5 Hz)/H-1 (δ_H_ 6.01), and between H-6 (δ_H_ 5.63, s)/H-7 (δ_H_ 2.49, t, *J* = 3.0 Hz), /H_ax_-8 (δ_H_ 2.58, dddd, *J* = 3.0, 7.2, 16.5 Hz) showed that the acetyl group and the benzoate ester at the 6 position were equatorial, while Me-14, Me-15 and the benzoate ester at the 9 position were axial. These results were the same as that of **3**. Taking into account biosynthetic relationship between **4** and **3**, **4** was elucidated as (1*R*,4*R*,5*S*,6*R*,7*R*,9*S*,10*R*)-1-acetoxy-6,9-dibenzoyloxydihydro-β-agarofuran-2-one because the only difference of **4** from **3** is the absence of one hydroxyl group at the 8 position. The findings of **3** and **4** have a special significance; up to date, there have been few of examples of natural dihydro-β-agarofuran ketones.

Compound **5** was isolated as a white solid, [α]_D_^15^ +43.34° (c 2.0, CH_3_OH). Its molecular formula was established as C_31_H_36_O_8_ by HR-MS (*m*/*z*: 559.2316 [M + Na]^+^) and ^13^C NMR data, which only has two additional hydrogen atoms than that of **4**. The IR spectrum showed the presence of hydroxyl (3530, 3063 cm^−1^), carbonyl (1718 cm^−1^) and aromatic (3063, 3016, 1602, 1585, 1480 cm^−1^) functionalities. The ^1^H and ^13^C NMR spectra of **5** were similar to those of **4**. Compared with **4**, **5** only had one additional oxygenated methine group and one less ketone carbonyl group.

The regiosubstitution of three ester groups were determined by HMBC experiment (Figure 2). The correlations between δ_C_ 167.0 (PhCO_2_–)/δ_H_ 5.73 (s, H-6), 8.07 (d, *J* = 7.4 Hz, benzoyl-H-2''), between δ_C_ 166.8 (PhCO_2_–)/δ_H_ 5.02 (1H, d, *J* = 7.2 Hz, H-9), /8.03 (d, *J* = 7.4 Hz, benzoyl-H-2'), and between δ_C_ 172.2 (MeCO_2_–)/δ_H_ 5.44 (1H, d, *J* = 3.4 Hz, H-1), /1.69 (s, CH_3_CO_2_–) suggested that one acetyl ester and two benzoyl esters were positioned at C-1, C-6 and C-9, respectively. In the ^1^H-^1^H COSY spectrum, the correlation between δ_H_ 4.42 (1H, dd, *J* = 3.0, 6.0 Hz, H-2)/δ_H_ 5.44 (1H, d, *J* = 3.4 Hz, H-1) revealed that the only hydroxyl group was at 2 position. The results above were further supported by the upfield chemical shifts of δ_H-1_, δ_H-3_, δ_H-4_, δ_C-1_, δ_C-3_ and δ_C-4_ in **5** relative to those in **4**. Thus, **5** was identified as the derivative of ketone carbonyl reduction of **4**.

NOE correlations between H-15 (δ_H_ 1.62, s)/H-14 (δ_H_ 1.32, d, *J* = 7.6 Hz), /H-6 (δ_H_ 5.73, s), /H-9 (δ_H_ 5.02, d, *J* = 6.8 Hz), between H-1 (δ_H_ 5.44, d, *J* = 3.4 Hz)/H-12 (δ_H_ 1.48, s), /H_ax_-3 (δ_H_ 2.34, dddd, *J* = 3.4, 6.0, 14.4 Hz) showed that the acetyl group at 1 position and the benzoate ester at 6 position were equatorial, while Me-14, Me-15 and the benzoate ester at 9 position were axial. NOE correlations between H-2 (δ_H_ 4.42, q, *J* = 3.0 Hz)/H-1 (δ_H_ 5.44, d, *J* = 3.4 Hz), /H_ax_-3 (δ_H_ 2.34, dddd, *J* = 3.4, 6.0, 14.4 Hz) showed that 2-OH was axial. As a result, compound **5** was elucidated as (1*R*,2*S*,4*R*,5*S*,6*R*,7*R*,9*S*,10*R*)-1-acetoxy-6,9-dibenzoyloxy-2-hydroxydihydro-β-agarofuran. 

Compound **6** was isolated as a white powder, [α]_D_^14.1^ +11.63° (c 2.0, CH_3_OH), was assigned the molecular formula C_33_H_38_O_10_, as deduced from HR-MS data (*m*/*z*: 617.2377 [M + Na]^+^) and ^13^C NMR data. Its IR absorptions were indicative of the presence of hydroxyl (3511 cm^−1^), aromatic (3062, 3032, 1602, 1585 cm^−1^) and ester (1746 and 1722 cm^−1^) groups. The ^1^H and ^13^C NMR, EI-MS, IR and UV spectra displayed the presence of two acetate esters, two benzoate esters and one hydroxyl group. The ^1^H and ^13^C NMR spectra of **6** (Table 1 and Table 2) were similar to those of **5** except for one additional oxygenated methine group [δ_C_ 75.2; δ_H_ 4.20 (1H, d, *J* = 3.2 Hz)], one additional acetate ester [δ_C_ 21.2, 172.0; δ_H_ 1.92 (s)] and one less methylene.

In the HMBC spectrum (Figure 2), the correlations between δ_H_ 5.42 (1H, q, *J* = 3.2 Hz, H-1), 1.49 (3H, s, AcO-1)/δ_C_ 171.7, between δ_H_ 5.49 (1H, dd, *J* = 3.2, 6.4 Hz, H-2), 1.92 (3H, s, AcO-2)/δ_C_ 172.0, between δ_H_ 6.30 (s, H-6), 7.97 (d, *J* = 7.4 Hz, benzoyl-H-2'')/δ_C_ 167.0 (PhCO_2_–), between δ_H_ 4.93 (1H, s, H-9), 7.91 (d, *J* = 7.4 Hz, benzoyl-H-2)/δ_C_ 166.8 (PhCO_2_–) revealed that two acetyl esters were located at C-1 and C-2, respectively, while two benzoyl esters were positioned at C-6 and C-9, respectively. The correlations between δ_H_ 4.93 (1H, s, H-9)/δ_C_ 75.2 (C-8) in the HMBC and the coupling between δ_H_ 2.46 (1H, d, *J* = 3.2 Hz, H-7) and δ_H_ 4.20 (1H, d, *J* = 3.2 Hz, H-8) in the ^1^H-^1^H COSY spectrum showed that the hydroxyl group was at 8 position.

Based on NOE effects (Figure 3) and the splitting patterns and coupling constants of H-1 (δ_H_ 5.52, d, *J* = 3.2 Hz), H-2 (δ_H_ 5.59, dd, *J* = 3.2, 6.4 Hz), H-6 (δ_H_ 6.40, s) and H-9 (δ_H_ 5.03, s), the stereochemical assignments of C-1, C-2, C-4, C-6 and C-9 were in agreement with the stereochemistry observed at these positions in **5**. NOE effect between H-8 (δ 4.30, d, *J* = 3.2 Hz)/H-12 [δ_H_ 1.36 (3H, s)] showed that the hydroxyl group at the 8 position was axial. Thus, **6** was elucidated as (1*R*,2*S*,4*R*,5*S*,6*R*,7*R*,8*R*,9*R*,10*R*)-1,2-diacetoxy-6,9-dibenzoyloxy-8-hydroxydihydro-β-agarofuran.

Compound **7** was isolated as a white powder, [α]_D_^15.2^ +8.50° (*c* 2.0, CH_3_OH), possessed a molecular formula C_31_H_36_O_9_, as deduced from HR-MS data (*m*/*z*: 575.2269 [M + Na]^+^) and ^13^C NMR data. The IR spectrum showed the presence of hydroxyl (3503 cm^−1^), carbonyl (1721 cm^−1^) and aromatic (3016, 3010, 1602, 1584 cm^−1^) functionalities. The ^1^H NMR, ^13^C NMR, EIMS, IR and UV spectra displayed the presence of one acetate ester, two benzoate esters and two hydroxyl groups. The ^1^H and ^13^C NMR spectra (Table 1 and Table 2) of **7**, including chemical shifts, splitting patterns and coupling constants, were very similar to those of **6** except for the lack of signals of one acetyl group. In the HMBC spectrum, the correlations between δ_H_ 5.20 (dd, *J* = 3.3, 6.0 Hz, H-2), 1.97 (s, Ac-H)/δ_C_ 172.6 (CH_3_CO_2_–), between δ_H_ 6.35 (s, H-6)/δ_C_ 166.8 (PhCO_2_–), and between δ_H_ 4.98 (s, H-9)/δ_C_ 166.7 (PhCO_2_–) showed that the acetyl ester and two benzoyl esters were located at C-2, C-6 and C-9, respectively. The splitting patterns and coupling constants between δ_H_ 4.45 (d, *J* = 3.3 Hz, H-1) and 5.25 (dd, *J* = 3.3, 6.0 Hz, H-2), and between δ_H_ 4.31 (d, *J* = 3.2 Hz, H-8) and 2.50 (d, *J* = 3.2 Hz, H-7) in the ^1^H NMR spectrum showed that two hydroxyl groups were located at C-1 and C-8, respectively. The above results were further confirmed by HMBC spectrum (Figure 1). In the NOESY spectrum, the NOEs were observed to be exactly the same as those of **6**, showing that **7** had the same stereochemistry as **6**. Thus, **7** was proposed as (1*R*,2*S*,4*R*,5*S*,6*R*,7*R*,8*R*,9*R*,10*S*)-2-acetoxy-6,9-dibenzoyloxy-1,8-dihydroxydihydro-β-agarofuran.

Compound **8** was isolated as a white solid [*α*]_D_^15^ +11.80° (*c* 2.0, CH_3_OH) and had the molecular formula C_31_H_36_O_9_, as deduced from HR-ESI-MS data (*m*/*z*: 575.2271 [M + Na]^+^), which was identical to that of **7**. The IR spectrum showed the presence of hydroxyl (3520 cm^−1^), aromatic (3063, 1602, 1585 cm^−1^) and carbonyl (1746, 1722 cm^−1^) groups. The ^1^H NMR (Table 2), ^13^C NMR (Table 1), EI-MS, IR and UV spectra displayed the presence of one acetate ester, two benzoate esters and two hydroxyl groups, in agreement with the case of **7**. The ^1^H and ^13^C NMR spectra of **8** (Table 1 and Table 2) were nearly the same as those of **7** except for the signals of H-1 and H-2, showing that **8** and **7** had the same dihydro-β-agarofuran core, and their structural difference only lied in the substituted patterns of C-1 and C-2. Based on the splitting patterns and coupling constants in the ^1^H NMR of **8**, the signals at δ_H_ 5.37 (1H, d, *J* = 3.2 Hz) and 4.42 (1H, d, *J* = 3.2, 6.0 Hz) were assigned as H-1 and H-2, respectively. In the HMBC spectrum, the correlations between δ_H_ 5.37 (H-1), 1.69 (3H, s, Ac-H)/δ_C_ 172.2 (CH_3_CO_2_–) showed that the acetoxy group was located at one site.

In the NOESY spectrum, the NOEs of **8** were observed to be the same as those of **7** (Figure 2). As the only difference of **8** from **7** was the transfer of the acetoxy group at the 2 site in **7** to the 1 site in **8**, this compound lastly was identified as having the same stereochemistry as **7**. Accordingly, the structure of **8** was deduced as (1*R*,2*S*,4*R*,5*S*,6*R*,7*R*,8*R*,9*R*,10*S*)-1-acetoxy-6,9-dibenzoyloxy-2,8-dihydroxydihydro-β-agarofuran.

To explore the potential bioactivities of the isolates from *P. wightiana* Wall, the compounds **2**–**8** were evaluated for *in vitro* cytotoxic properties against HL-60, SMMC-7721, A549, MCF-7 and SW480 cell lines by MTT assays. Because of poor solubility, compound **1** was not tested for cytotoxicity. The cytotoxicity data are shown in Table 3. The anticancer agent *cis*-diaminodichloroplatinum was used as a positive control.

All the test compounds showed the definite activities in varying degrees against all tested cell lines at the concentration of 40 μM. For most of the cell lines, compounds **3**, **5**–**8** exhibited moderate activities with IC_50_ values ranging from 11.8 to 30.6 μM, while **2** and **4** displayed poor activities with IC_50_ values >40 μM in most cases. It was worth noting that **4** showed higher activity against A-549 (IC_50_ = 17.4 μM). For each of the tested cell lines, **5**, **6** or **7** were the most cytotoxic.

**Table 2:** ^1^H NMR data of compounds **3**–**8** (400 MHz; **3**, **4**, CD_3_Cl_3_; **5**–**8**, CD_3_OD).

Number	3, δ_H_^a^, *J* in Hz	4, δ_H_^a^, *J* in Hz	5, δ_H_, *J* in Hz	6, δ_H_, *J* in Hz	7, δ_H_, *J* in Hz	8, δ_H_, *J* in Hz
1	5.98, s	6.01, s	5.44, d, 3.4	5.42, d, 3.2	4.45, d, 3.3	5.37, d, 3.2
2			4.42, dd, 3.0, 6.0	5.49, dd, 3.2, 6.4	5.20, dd, 3.3, 6.0	4.42, dd, 3.2, 6.0
3_eq_	2.29, d, 12.8	2.27, d, 12.5	1.88, br d, 14.4	1.75, br d, 15.2	1.82, br d, 15.0	1.86, br d, 14.8
3_ax_	3.39, dd, 7.4, 12.8	3.40, dd, 7.3, 12.5	2.34, dddd, 3.4, 6.0, 14.4	2.35, dddd, 3.6, 6.4, 15.1	2.31, dddd, 3.3, 6.0, 15.0	2.33, dddd, 3.2, 6.0, 14.8
4	2.99–3.05 quintet-like	2.98–3.04 quintet-like	2.48–2.55 quintet-like	2.43–2.51 quintet-like	2.40–2.48 quintet-like	2.46–2.53 quintet-like
6	6.27, s	5.63, s	5.73, s	6.30, s	6.35, s	6.40, s
7	2.72, s	2.49, t, 3.0	2.41, t-like, 2.8	2.46, d, 3.2	2.50, d, 3.2	2.53, d, 3.2
8eq	4.48, s	2.33, dd, 3.0, 16.5	2.21, dd, 3.0, 16.4	4.20, d, 3.2	4.31, d, 3.2	4.29, d, 3.2
8ax	-	2.58, dddd, 3.0, 7.2, 16.5	2.58, dddd, 3.2, 6.8, 16.4	-	-	-
9	4.97, s	5.08, d, 7.2	5.02, d, 6.8	4.93, s	4.98, s	5.04, s
12	1.50, s	1.55, s	1.45, s	1.36, s	1.39, s	1.44, s
13	1.59, s	1.55, s	1.48, s	1.45, s	1.48, s	1.54, s
14	1.06, d, 7.5	1.02, d, 7.5	1.32, d, 7.6	1.18, d, 7.6	1.15, d, 7.6	1.30, d, 7.6
15	1.46, s	1.34, s	1.62, s	1.60, s	1.55, s	1.75, s
AcO-1	1.71, s	1.73, s	1.69, s	1.49, s	-	1.69, s
AcO-2	-	-	-	1.92, s	1.97, s	-
2'/6'	8.03, d, 8.0	8.05, d, 7.4	8.03, d, 7.4	7.91, d, 7.4	8.03, d, 7.4	8.02, d, 7.3
3'/5'	7.46, t, 8.0	7.45, t, 7.4	7.47, d, 7.4	7.37, t, 7.4	7.44, d, 7.4	7.47, d, 7.3
4'	7.59, t, 8.0	7.57, t, 7.4	7.60, d, 7.4	7.51, t, 7.4	7.55, t, 7.4	7.61, d, 7.3
2''/6''	8.05, d, 8.0	8.06, d, 7.4	8.07, d, 7.4	7.97, d, 7.4	8.01, d, 7.4	8.06, d, 7.3
3''/5''	7.49, t, 8.0	7.50, t, 7.4	7.54, d, 7.4	7.44, t, 7.4	7.47, d, 7.4	7.54, d, 7.3
4''	7.62, t, 8.0	7.63, t, 7.4	7.66, d, 7.4	7.56, t, 7.4	7.59, t, 7.4	7.66, d, 7.3

^a^ The data were acquired at 500 MHz.

**Table 3:** Cytotoxicities of compounds **2**–**8** against five cancer cell lines.

Compound	IC_50_ (μM)				
	HL-60	SMMC-7721	A-549	MCF-7	SW480
**2**	>40	>40	>40	>40	>40
**3**	17.6	20.8	24.1	23.0	>40
**4**	>40	35.9	17.4	>40	>40
**5**	15.2	18.6	21.2	17.1	12.8
**6**	17.6	18.6	17.2	21.4	30.1
**7**	16.0	29.3	23.1	16.9	11.8
**8**	18.4	>40	30.6	21.6	34.2
DDP ^a^	3.1	10.2	9.1	17.5	12.0

^a^ DDP: *cis*-diaminodichloroplatinum which was used as a positive control.

Preliminary analysis of the structure–activity relationship revealed that the compounds with hydroxyl groups (**3**, **5**–**8**) had greater cytotoxicity than the compounds without hydroxyl groups (**2**, **4**). The reduction of ketone carbonyl group in **4** to hydroxyl group such as **5** or the introduction of 8-OH into **4** such as **3** led to a significant improvement of activity, except against A-549 cells, showing that 2-OH and 8-OH were beneficial for the improvement of activity against most of the cell lines, and the presence of 2-carbonyl group was able to increase activity against A-549 cells. However, the 2,8-dihydroxy compound **8** did not demonstrate higher activity than 2-hydroxy compound **5** or 8-hydroxy compound **3**. The higher activity of **7** than **8** showed that the combination of 1,8-dihydroxy groups led to higher activity than that of 2,8-dihydroxy groups. In addition, the 1,8-dihydroxy compound **7** demonstrated higher activity than the corresponding 1-acetoxy-8-hydroxy compound **6** against HL-60, MCF-7 and SW480 but lower activity against SMMC-7721 and A-549.

## 3. Experimental Section

### 3.1. General

Melting points were determined on an XT-4 micro-melting point apparatus and were uncorrected. Optical rotations were measured with an Autopol V instrument (Rudolph Research Analytical, Hachettstown, NJ, USA). Infrared (IR) spectra were recorded in wave numbers (cm^−1^) on a Bruker TENSOR 27 transform infrared spectrophotometer (Bruker Daltonics Inc., Bremen, Germany) with KBr disks. ^1^H NMR spectra at 400 or 500 MHz and ^13^C NMR spectra at 100 or 125 MHz were recorded with a Bruker AVANCE III spectrometer (Bruker Daltonics Inc., Bremen, Germany) with TMS as internal standard. Coupling constants were reported in hertz (Hz). Electrospray ionization (ESIMS) was measured on a 4000 QTRAP LC-MS instrument (Thermo Finnigan Co., Waltham, MA, USA). MS spectra were obtained from a 4000 QTRAP LC-MS in MS mode. High-resolution mass spectra (HR-MS) were determined on a Thermo LTQ XL Orbitrap instrument (Thermo Finnigan Co., Waltham, MA, USA). Analytic Preparative HPLC was carried out using a Shimadzu LC-20AD instrument (Shimadzu Corporation, Kyoto, Japan) equipped with an Agilent ZORBAX Eclipse Plus C_18_ column (5 μm, 4.6 mm × 150 mm) and SPD-20A UV/vis detector (Shimadzu Corporation, Kyoto, Japan), and using methanol-water (70:30) as a mobile phase. Preparative HPLC was carried out using a Shimadzu LC-8A instrument (Shimadzu Corporation, Kyoto, Japan) equipped with a SHIM-PACK PRC-ODS column (5 μM, 20 mm × 250 mm) and SPD-M10Avp photodiode array detector and using methanol-water (80:20) as a mobile phase. Column chromatographic (CC) separations were performed using silica gel H60 (300–400 mesh) (Qingdao Marine Chemical Ltd., Qingdao, China), and Sephadex LH-20 (Pharmacia Biotech AB, Uppsala, Sweden) as packing materials.

### 3.2. Experimental Procedures

#### 3.2.1. Plant Material

The entire plants of *P. wightiana* were collected from Qinling Mountains, Shaanxi province, China, in June 2013, and identified by Professor Fang Miao, a co-author of this paper. A voucher specimen (No. 20061202) is deposited in botanic specimen center of Northwest A&F University, Yangling, China.

#### 3.2.2. Extraction and Isolation

Powdered and air-dried materials of *Parnassia wightiana* Wall and Wight and Arn were successively extracted with petroleum ether (bp 60–90 °C) at room temperature three times (3 × 72 h). After removal of solvent *in vacuo*, the black viscous residue (43.5 g) was treated with methanol to remove insoluble black oily deposits. The methanol-soluble fraction was subjected to silica gel column chromatography eluting with petroleum ether-ethyl acetate (stepwise, 1:0, 50:1, 30:1, 10:1, 5:1, 2:1, 1:1, 0:1, each 500 mL) and finally methanol (500 mL). Nine fractions (F_1_–F_9_) were obtained. Compound **1** was precipitated from F_7_ corresponding to the eluent of petroleum ether-ethyl acetate (1:1), and recrystallized in ethyl acetate to afford pure **1** (250 mg). The mother solution after removal of **1** was evaporated to dryness. The resulting residue (8.23 g) was chromatographed on a silica gel column eluting with petroleum ether-ethyl acetate (stepwise, 2:1, 2:1.5, 1:1, 1:2, 1:4, 0:1, each 500 mL) to afford six subfractions (F_71_–F_76_) by HPLC analysis. F_73_ (0.54 g) was separated by preparative HPLC using a gradient of MeOH-H_2_O (80:20 over 40 min, 10 mL·min^−^^1^) to yield **5** (76.8 mg), **6** (25.4 mg) and **3** (180.7 mg). The same steps as described for F_73_ were used to separate F_74_ (0.784 g), and yielded **8** (156.4 mg) and **7** (84.4 mg), and F_6_ (7.02 g) to afford **4** (14.0 mg), **2** (55.8 mg) and **3** (83.7 mg).

##### (+)-(1*R*,2*S*,4*R*,5*S*,6*R*,7*R*,8*R*,9*R*,10*S*)-1,2,6,8,15-Pentaacetoxy-9-benzoyloxydihydro-β-agarofuran (**1**)

Compound **1**: a colorless crystal; mp 236–238 °C; [*α*]_D_^17^ 0.923° (*c* 2.0, AcOEt); Its [α]_D_^t^ value was not provided in the literature [14]. UV (MeOH) λ_max_ (log *ε*) 244 (3.69), 276 (3.01), 284 (2.92) nm; IR ν_max_ (KBr) 3061, 3016, 2978, 2936, 2893, 1742, 1716, 1602, 1586, 1490, 1453, 1433, 1371, 1318, 1277, 1235, 1158, 1147, 1100, 1085, 1048, 1026, 982, 959, 932, 914, 887, 826, 713, 601 cm^−1^; ^1^H NMR (400 MHz, CDCl_3_) (ppm): δ 8.00 (2H, d, *J* = 7.6 Hz, H-2', H-6'), 7.58 (1H, t, *J* = 7.6 Hz, H-4'), 7.44 (2H, t, *J* = 7.6 Hz, H-3', H-5'), 6.39 (1H, s, H-6), 2.38 (1H, d, *J* = 3.0 Hz, H-7), 5.71 (1H, d, *J* = 3.8 Hz, H-1), 5.60 (1H, dd, *J* = 4.0, 6.4 Hz, H-2), 5.52 (1H, s, H-9), 5.28 (1H, d, *J* = 3.0 Hz, H-8), 1.78 (1H, m, H-3eq), 5.10 (1H, d, *J* = 12.8 Hz, H-15a), 4.54 (1H, d, *J* = 12.8 Hz, H-15b), 2.72 (3H, s, AcO-15), 2.50 (1H, dddd, *J* = 4.0, 6.4, 10.4 Hz, H-3ax), 2.37–2.44 (1H, quintet-like, H-4), 2.20 (3H, s, AcO-8), 2.10 (3H, s, AcO-6), 2.08 (3H, s, AcO-2), 1.57 (3H, s, Me-12), 1.46 (3H, s, AcO-1), 1.43 (3H, s, Me-13), 1.17 (3H, d, *J* = 7.6 Hz, Me-14); ^13^C NMR (100 MHz, CDCl_3_) (ppm): δ 170.5 (CH_3_CO_2_-15), 169.9 (CH_3_CO_2_-2), 169.8 (CH_3_CO_2_-6), 169.6 (CH_3_CO_2_-8), 169.3 (CH_3_CO_2_-1), 164.6 (C_6_H_5_CO_2_-5), 133.8 (C-4'), 130.2 (C-2', C-6'), 128.5 (C-1'), 128.4 (C-3', C-5'), 89.8 (C-5), 81.5 (C-11), 76.9 (C-8), 74.9 (C-6), 74.0 (C-9), 71.6 (C-1), 69.1 (C-2), 65.5 (C-15), 53.1 (C-7), 52.6 (C-10), 32.7 (C-4), 31.0 (C-3), 30.2 (C-13), 25.7 (C-12), 21.3 (CH_3_CO_2_-8), 21.3 (CH_3_CO_2_-15), 21.3 (CH_3_CO_2_-6), 21.2 (CH_3_CO_2_-2), 20.4 (CH_3_CO_2_-1), 16.9 (C-14). The NMR data were in agreement with the literature values [13]. (+)-HR-ESI-MS *m*/*z* 655.2378 [M + Na]^+^ (calcd. for C_32_H_40_NaO_13_, 655.2367).

Crystallographic Data and X-ray Structure Analysis of **1**. A colorless crystal (0.25 × 0.20 × 0.18) of **1** was grown by slow evaporation in AcOEt solution. Diffraction intensity data were acquired with a CCD area detector with graphite monochromated Cu Kα radiation (λ = 1.54184 Å). Crystal data for 1: C_32_H_40_O_13_, Mr = 632.64, trigonal, space group P3_1_, Z = 3, *a* = 15.04493(18), *b* = 15.04493(18), *c* = 12.5329(2) Å, α = 90.00°, β = 90.00°, γ = 120.00°, *V* = 2456.76(6) Å^3^, *T* = 291(2) K, μ (Cu Kα) = 0.837 mm^−1^, 21,172 reflections measured, 6040 independent reflections (*R*_int_ = 0.0191), *S* = 1.029. The final *R*_1_ values were 0.0361 and *R*_w_ = 0.098 (*I* > 2σ (*I*)). The final *R*_1_ values were 0.0377 and *R*_w_ = 0.0997 (for all data). Flack parameter = −0.06(12). Crystallographic data for 1 have been deposited with the Cambridge Crystallographic Data Centre (deposition number CCDC 979627). Copies of the data can be obtained, free of charge, on application to the Director, CCDC, 12 Union Road, Cambridge CB21EZ, UK.

##### (+)-(1*R*,2*S*,4*R*,5*S*,6*R*,7*R*,9*S*,10*R*)-1,2,6,15-Tetraacetoxy-9-benzoyloxydihydro-β-agarofuran (**2**)

Compound **2**: a white solid; [α]_D_^21^ +44.50 (*c* 0.517, CHCl_3_); lit. [*α*]_D_^14^ +44.49° (*c* 0.517, CHCl_3_) [14a]; UV (CHCl_3_) λ_max_ (log *ε*) 244 (3.68), 276 (3.12), 283 (3.04) nm; IR ν_max_ (KBr) 3063, 2972, 2935, 1748, 1716, 1603, 1585, 1453, 1438, 1369, 1279, 1246, 1143, 1092, 1040, 1024, 876, 714 cm^−1^; ^1^H NMR (500 MHz, CDCl_3_) (ppm): δ 8.02 (2H, d, *J* = 7.4 Hz), 7.43–7.45 (2H, m, C_6_H_5_CO), 7.56 (1H, t, *J* = 7.0 Hz), 5.97 (1H, s, H-5), 5.71 (1H, d, *J* = 3.0 Hz, H-1), 5.41 (1H, d, *J* = 7.1 Hz, H-9), 5.07 (1H, d, *J* = 12.7 Hz, H-13a), 4.37 (1H, d, *J* = 12.8 Hz, H-13b), 2.50–2.54 (1H, m, H-8a), 2.45–2.47 (1H, m, H-3a), 2.38–2.40 (1H, m, H-4), 2.25 (1H, d, *J* = 2.8, H-7), 2.19 (1H, t, *J* = 12.1 Hz, H-8b), 1.77–7.80 (1H, m, H-3b), 1.45 (3H, s, H-14), 1.54, 2.08, 2.10, 2.25 (each 3H, s, 4 × Ac), 1.43 (3H, s, H-15), 1.18 (3H, d, *J* = 7.6 Hz, H-12); ^13^C NMR (125 MHz, CDCl_3_) (ppm): δ 170.5, 170.0, 169.9, 169.3, (4 × Ac), 165.3 (C_6_H_5_CO), 133.5, 130.1, 129.1, 128.3, 89.2 (C-5), 82.7 (C-11), 78.1 (C-6), 71.5 (C-1), 69.51 (C-9), 69.45 (C-2), 65.5 (C-13), 53.3 (C-10), 48.8 (C-7), 34.9 (C-8), 33.1 (C-4), 30.8 (C-3), 30.3 (C-14), 25.9 (C-15), 21.35, 21.29, 21.2, 20.3, 17.8 (C-12); The NMR data were in agreement with the literature values [14]. (+)-EIMS *m*/*z*: 574 [M]^+^, 559 [M − Me]^+^, 531[M − Ac]^+^, 105 [C_6_H_5_CO]^+^, 43 [Ac]^+^; (+)-ESI-MS *m*/*z*: 597 [M + Na]^+^, 539 [M + Na-Ac-Me]^+^.

##### (+)-(1*R*,4*R*,5*S*,6*R*,7*R*,8*R*,9*R*,10*R*)-1-Acetoxy-6,9-dibenzoyloxy-8-hydroxy dihydro-β-agarofuran-2-one (**3**)

Compound **3**: a white solid; mp 133–135 °C; [*α*]_D_^21^ +10.45° (*c* 0.331, CHCl_3 _); UV (MeOH) λ_max_ (log ε) 202 (4.58), 232 (4.61), 274 (3.60), 280 (3.54) nm; IR ν_max_ (KBr) 3493, 3063, 2970, 2930, 1752, 1724, 1601, 1452, 1386, 1370, 1271, 1231, 1107, 1070, 1025, 712 cm^−1^; ^13^C NMR and ^1^H NMR data, see Table 1 and Table 2. (+)-EI-MS *m*/*z*: 535 [M − Me]^+^, 105 [C_6_H_5_CO]^+^, 77 [C_6_H_5_]^+^, 43 [Ac]^+^; (+)-ESI-MS *m*/*z*: 551 [M + H]^+^, 573 [M + Na]^+^; (+)-HR-ESI-MS *m*/*z*: 573.2107 [M + Na]^+^ (calcd. for C_31_H_34_NaO_9_, 573.2101).

##### (+)-(1*R*,4*R*,5*S*,6*R*,7*R*,9*S*,10*R*)-1-Acetoxy-6,9-dibenzoyloxydihydro-β-agarofuran-2-one (**4**)

Compound **4**: a white powder; mp 120–122 °C; [α]_D_^21^ +13.23° (*c* 0.410, CHCl_3 _); UV (CHCl_3_) λ_max_ (log ε) 244 (4.05), 275 (3.51), 283 (3.43) nm; IR ν_max_ (KBr) 3062, 2966, 2930, 1753, 1720, 1601, 1451, 1387, 1314, 1273, 1229, 1105, 1070, 1025, 712 cm^−1^; ^13^C NMR and ^1^H NMR data, see Table 1 and Table 2; (+)-EIMS *m*/*z*: 534 [M]^+^, 105 [C_6_H_5_CO]^+^, 77 [C_6_H_5_]^+^, 43 [Ac]^+^; (+)-HR-ESI-MS *m*/*z*: 557.2144 [M + Na]^+^ (calcd. for C_31_H_34_NaO_8_, 557.2151).

##### (+)-(1*R*,2*S*, 4*R*,5*S*,6*R*,7*R*,9*S*,10*R*)-1-Acetoxy-6,9-dibenzoyloxy-2-hydroxydihydro-β-agarofuran (**5**)

Compound **5**: a white solid; mp 110–112 °C; [α]_D_^17^ +43.34° (*c* 2.0, CH_3_OH); UV (MeOH) λ_max_ (log ε) 202 (4.75), 231 (4.87), 274 (3.69), 281 (3.60) nm; IR ν_max_ (KBr) 3530 (br), 3063, 3016, 2970, 2928, 1718, 1602, 1585, 1480, 1452, 1387, 1368, 1276, 1176, 1149, 1107, 1069, 1025, 1014, 969, 712 cm^−1^; ^13^C NMR and ^1^H NMR data, see Table 1 and Table 2; (+)-EIMS *m*/*z*: 536 [M]^+^, 105 [C_6_H_5_CO]^+^, 77 [C_6_H_5_]^+^, 43 [Ac]^+^; (+)-HR-ESI-MS *m*/*z* 559.2316 [M + Na]^+^ (calcd. for C_31_H_36_NaO_8_, 559.2308).

##### (+)-(1*R*,2*S*,4*R*,5*S*,6*R*,7*R*,8*R*,9*R*,10*R*)-1,2-Diacetoxy-6,9-dibenzoyloxy-8-hydroxydihydro-β-agarofuran (**6**)

Compound **6**: a white powder; mp 129–131 °C; [α]_D_^14.1^ +11.63° (*c* 2.0, CH_3_OH); UV (MeOH) λ_max_ (log ε) 202 (4.68), 231 (4.68), 274 (3.61), 280 (3.54) nm; IR *ν*_max_ (KBr) 3511 (br), 3062, 3032, 2972, 2930, 1746, 1722, 1602, 1585, 1452, 1387, 1367, 1273, 1178, 1107, 1069, 1024, 983, 892, 712 cm^−1^; ^13^C NMR and ^1^H NMR data, see Table 1 and Table 2; (+)-EIMS *m*/*z*: 579 [M − Me]^+^, 105 [C_6_H_5_CO]^+^, 77 [C_6_H_5_]^+^, 43 [Ac]^+^; (+)-HR-ESI-MS *m*/*z*: 617.2377 [M + Na]^+^ (calcd. for C_33_H_38_NaO_10_, 617.2363).

##### (+)-(1*R*,2*S*,4*R*,5*S*,6*R*,7*R*,8*R*,9*R*,10*S*)-2-Acetoxy-6,9-dibenzoyloxy-1,8-dihydroxydihydro-β-agarofuran (**7**)

Compound **7**: a white powder; mp 128–130 °C; [α]_D_^15.2^ +8.50° (*c* 2.0, CH_3_OH); UV (MeOH) λ_max_ (log ε) 202 (4.66), 231 (4.70), 273 (3.61), 280 (3.54) nm; IR ν_max_ (KBr) 3503, 3062, 3034, 3010, 2970, 2930, 1721, 1602, 1584, 1452, 1386, 1367, 1316, 1274, 1231, 1177, 1144, 1109, 1069, 1027, 982, 712 cm^−1^; ^13^C NMR and ^1^H NMR data, see Table 1 and Table 2; (+)-EIMS *m*/*z*: 537 [M − Me]^+^, 430 [M − C_6_H_5_CO_2_H]^+^, 105 [C_6_H_5_CO]^+^, 77 [C_6_H_5_]^+^, 43 [Ac]^+^; (+)-HR-MS *m*/*z*: 575.2269 [M + Na]^+^ (calcd. for C_31_H_36_NaO_9_, 575.2257).

##### (+)-(1*R*,2*S*,4*R*,5*S*,6*R*,7*R*,8*R*,9*R*,10*S*)-1-Acetoxy-6,9-dibenz oyloxy-2,8-dihydroxydihydro-β-agarofuran (**8**)

Compound **8**: a white solid; mp 134–136 °C; [α]_D_^15^ +11.80° (*c* 2.0, CH_3_OH); UV (MeOH) λ_max_ (log ε) 202 (4.76), 231 (4.81), 274 (3.86), 313 (3.25) nm; IR ν_max_ (KBr) 3520 (br), 3063, 2972, 2929, 1746, 1722, 1602, 1585, 1452, 1370, 1316, 1274, 1178, 1146, 1097, 1070, 1027, 1007, 982, 712 cm^−1^; ^13^C NMR and ^1^H NMR data, see Table 1 and Table 2; (+)-EI-MS *m*/*z*: 105[C_6_H_5_CO]^+^, 43[Ac]^+^; (+)-HR-ESI-MS *m*/*z*: 575.2271 [M + Na]^+^ (calcd. for C_31_H_36_NaO_9_, 575.2257).

#### 3.2.3. Cytotoxicity Assay

The cytotoxicity assay was performed according to the modified MTT colorimetric method [17]. HL-60 cells (Human promyelocytic leukemia cell line), SMMC-7721 cells (human hepatocellular carcinoma cell line), A549 cells (adenocarcinomic human alveolar basal epithelial cell line), MCF-7 cells (human breast adenocarcinoma cell line) and SW480 cells (Human colon adenocarcinoma cell line) were used as tested cells. All cell lines were cultured at 37 °C under a humidified atmosphere of 5% CO_2_ in RPMI 1640 medium supplemented with 10% fetal serum and dispersed in replicate 96-well plates. Eight concentrations of test compounds (dissolved in 0.5% DMSO) encompassing a 128-fold range were added to each cell line. After 48 h exposure to the compounds, cells’ viability was determined by the [3-(4,5-dimethylthiazol-2-yl)-5(3-carboxymethoxyphenyl)-2-(4-sulfopheny)-2H-tetrazolium] (MTS) cytotoxicity assay by measuring the absorbance at 490 nm with a microplate spectrophotometer. Each test was performed in triplicate. The IC_50_ was defined as the concentration of the test compound resulting in a 50% reduction of absorbance compared to untreated cells in the MTT assay. The anticancer agent DDP (*cis*-diamminedichloroplatinum) and 0.5% DMSO were used as the positive control and solvent control, respectively. 

## 4. Conclusions

In conclusion, we isolated five new and three known dihydro-β-agarofuran sesquiterpene polyesters from *Parnassia wightiana* and evaluated their cytotoxicities *in vitro* against five cancer cell lines. The structures of all the compounds were established through spectroscopic analysis, and their absolute configurations were established by X-ray diffraction analysis and biogenetic means. For most of tested cell lines, **3**, **5**, **6** and **7** exhibited moderate activity with IC_50_ values of 11.8–30.1 μM. In addition, the structure–activity relationship was discussed as well.
